# First record of the genus *Fagineura* Vikberg & Zinovjev (Hymenoptera, Tenthredinidae) with descriptions of two new species from China

**DOI:** 10.3897/zookeys.829.30086

**Published:** 2019-03-11

**Authors:** Mengmeng Liu, Zejian Li, Meicai Wei

**Affiliations:** 1 Lab of Insect Systematics and Evolutionary Biology, Central South University of Forestry and Technology, 498 South Shaoshan Road, Changsha 410004, Hunan, China Central South University of Forestry and Technology Changsha China; 2 Lishui Academy of Forestry, Lishui 323000, Zhejiang, China Lishui Academy of Forestry Zhejiang China; 3 College of Life Science, Jiangxi Normal University, Nanchang 330026, Jiangxi, China Jiangxi Normal University Nanchang China

**Keywords:** COI, key, NaK, Nematinae, sawfly, Symphyta, taxonomy

## Abstract

*Fagineura* Vikberg & Zinovjev, 2000 is recorded from China for the first time. Two species of *Fagineura* are described as new, *F.flactoserrula***sp. n.** and *F.xanthosoma***sp. n.** A key to the species of *Fagineura* worldwide is provided, now including four species. In addition, a simple phylogenetic analysis of *Fagineura* species is provided, based on sequences of the COI and NaK genes.

## Introduction

*Fagineura* Vikberg & Zinovjev, 2000 (Shinohara et al. 2000) is a very small genus of the subfamily Nematinae (Tenthredinidae). Until now, there are only two known species in the world (Taeger et al. 2010), namely *F.crenativora* Vikberg & Zinovjev, 2000 (type species) and *F.quercivora* Togashi, 2006, both of which are distributed in Japan. In a study of Nematinae from China, two species of *Fagineura* were found that are different from the two known species in Japan, and they are described herein as new species. Additionally, the genus *Fagineura* is recorded as a new genus in China. The two species are described and illustrated, a key to the known species of *Fagineura* worldwide is provided, and a simple phylogenetic analysis based on DNA sequence data from two genes (COI and NaK) is provided.

## Materials and methods

### Imaging, terminology, deposition of material

The specimens were examined with a Motic-SMZ-171 stereomicroscope. Images of the imagines were taken with a Nikon D700 digital camera and a Leica Z16APO separately. The genitalia were examined with a Motic BA410E microscope, and images of the genitalia were taken with Motic Moticam Pro 285A. The series of images produced were montaged using Helicon Focus (HeliconSoft, Kharkiv, Ukraine) and further processed with Adobe Photoshop CS 11.0.

Morphological descriptions of the new species are based on the holotypes. The terminology of genitalia follows Ross (1945) and that of general morphology follows Viitasaari (2002). For a few terms, including middle fovea, lateral fovea, and lateral walls, we follow Takeuchi (1952).

Specimens examined in this study are deposited in the Central South University of Forestry and Technology, Changsha (CSCS), China, including all holotypes and paratypes of the two new species.

### Phylogenetic analyses

DNA was extracted from adult samples stored in 99.5% ethanol at -20 °C by using the DNeasy Tissue Kit (Qiagen, Valencia, CA). Sequence data were obtained from the mitochondrial gene cytochrome oxidase I (COI; 810 bp) and the nuclear gene sodium-potassium adenosine triphosphatase (NaK; 952 bp). PCR amplification of COI and NaK were performed as described previously (Normark et al. 1999; Nyman et al. 2006; Leppänen et al. 2012). New sequences have been deposited in GenBank under accession numbers MH544099–MH544102. COI and NaK sequences of Nematinae species used in previous phylogenetic analyses are available in GenBank, and their accession numbers and references are shown in Table 1.

**Table 1.** COI and NaK sequences of Nematinae species analyzed in this work.

Species Name	GenBank Accession Number		Reference
	COI	NaK	
* Anoplonyx apicalis *	DQ302172	KJ434879	Nyman et al. (2006), Prous et al. (2014)
* Caulocampus acericaulis *	DQ302182	KJ434873	Nyman et al. (2006), Prous et al. (2014)
* Craterocercus fraternalis *	DQ302170	KJ434878	Nyman et al. (2006), Prous et al. (2014)
* Endophytus anemones *	DQ302186	KJ434900	Nyman et al. (2006), Prous et al. (2014)
* Euura amerinae *	KJ434923	KJ434915	Prous et al. (2014)
* Euura annulata *	DQ302195	KJ434876	Nyman et al. (2006), Prous et al. (2014)
* Euura dimmockii *	DQ302192	KJ434885	Nyman et al. (2006), Prous et al. (2014)
* Euura dolichura *	DQ302213	KJ434858	Nyman et al. (2006), Prous et al. (2014)
* Euura herbaceae *	DQ302217	KJ434860	Nyman et al. (2006), Prous et al. (2014)
* Euura imperfecta *	DQ302210	KJ434883	Nyman et al. (2006), Prous et al. (2014)
* Euura lanatae *	DQ302219	KJ434907	Nyman et al. (2006), Prous et al. (2014)
* Euura leucapsis *	KJ434922	KJ434909	Prous et al. (2014)
* Euura lipovskyi *	DQ302206	KJ434892	Nyman et al. (2006), Prous et al. (2014)
* Euura melanaspis *	DQ302205	KJ434863	Nyman et al. (2006), Prous et al. (2014)
* Euura miliaris *	DQ302207	KJ434895	Nyman et al. (2006), Prous et al. (2014)
* Euura montana *	DQ302193	KJ434868	Nyman et al. (2006), Prous et al. (2014)
* Euura pumilio *	DQ302190	KJ434882	Nyman et al. (2006), Prous et al. (2014)
* Euura ribesii *	DQ302208	KJ434871	Nyman et al. (2006), Prous et al. (2014)
* Euura saliciscinereae *	DQ302216	KJ434859	Nyman et al. (2006), Prous et al. (2014)
* Euura scutellata *	DQ302191	KJ434866	Nyman et al. (2006), Prous et al. (2014)
* Euura venusta *	DQ302220	KJ434862	Nyman et al. (2006), Prous et al. (2014)
* Fagineura crenativora *	DQ302233	KJ434899	Nyman et al. (2006), Prous et al. (2014)
* Fagineura flactoserrula *	MH544099	MH544101	This work
* Fagineura xanthosoma *	MH544100	MH544102	This work
* Fallocampus americanus *	DQ302178	KJ434903	Nyman et al. (2006), Prous et al. (2014)
* Kerita fidala *	KJ434918	KJ434826	Prous et al. (2014)
* Mesoneura opaca *	DQ302169	KJ434877	Nyman et al. (2006), Prous et al. (2014)
* Mesoneura shishikuensis *	KY698135	KY698259	Prous et al. (2017)
* Nematus erythrogaster *	KJ434917	KJ434818	Prous et al. (2014)
* Nematus princeps *	KJ434921	KJ434865	Prous et al. (2014)
* Nematus septentrionalis *	DQ302197	KJ434875	Nyman et al. (2006), Prous et al. (2014)
* Nematus tulunensis *	DQ302209	KJ434872	Nyman et al. (2006), Prous et al. (2014)
* Priophorus pallipes *	DQ302167	KJ434890	Nyman et al. (2006), Prous et al. (2014)
* Pristiphora abbreviata *	KJ434920	KJ434848	Prous et al. (2014)
* Pristiphora abietina *	DQ302227	KJ434869	Nyman et al. (2006), Prous et al. (2014)
* Pristiphora alpestris *	DQ302228	KJ434897	Nyman et al. (2006), Prous et al. (2014)
* Pristiphora coactula *	DQ302229	KJ434870	Nyman et al. (2006), Prous et al. (2014)
* Pristiphora ferruginosa *	DQ302188	KJ434893	Nyman et al. (2006), Prous et al. (2014)
* Pristiphora geniculata *	DQ302225	KJ434898	Nyman et al. (2006), Prous et al. (2014)
* Pristiphora litura *	DQ302231	KJ434894	Nyman et al. (2006), Prous et al. (2014)
* Pristiphora monogyniae *	DQ302223	KJ434880	Nyman et al. (2006), Prous et al. (2014)
* Pseudodineura mentiens *	KJ434919	KJ434841	Prous et al. (2014)

The data of each newly sequenced sample are as follows:

*Fagineuraflactoserrula* sp. n.: Paratype, 1♀, China, Hubei Province, Yichang City, Shennongjia Mountain, Yinyuhe, 31°34'00"N, 110°20'22"E, 2100 m, 16 May 2012, leg. Zejian Li; the GenBank Accession Numbers of COI and NaK are MH544099 and MH544101, respectively.

*F.xanthosoma* sp. n.: Paratype, 1♀, China, Hubei Province, Yichang City, Shennongjia Mountain, Yinyuhe, 31°34'00"N, 110°20'22"E, 2100 m, 17 May 2012, leg. Zejian Li; the GenBank Accession Numbers of COI and NaK are MH544100 and MH544102, respectively.

The final two-gene alignment is 1762 base pairs long and contains 42 specimens from 13 genera. The genetic distances among species were calculated based on Kimura 2-parameter model of the two genes in Mega 7 (Kumar et al. 2016). Bayesian phylogenetic analyses were performed in MrBayes 3.2.6 (Ronquist et al. 2012). The dataset was not partitioned, and the best-fitting DNA substitution model for the two-gene alignment was selected using jModelTest 2.1.7 (Darriba et al. 2012), which uses PhyML (Guindon and Gascuel 2003) for likelihood calculations. Model selection was done by selecting among 11 substitution schemes (including 88 different models) on the basis of the Akaike Information Criterion (AIC).

Abbreviations used in the text and illustrations are as follows:

**OCL** The distance between a lateral ocellus and the occipital carina, or the hind margin of the head where this carina would be if it were developed (Benson 1954).

**OOL** The distance between an eye and a lateral ocellus.

**POL** The distance between the mesal margins of the 2 lateral ocelli.

## Results

### Taxonomy

#### Fagineura

Taxon classificationAnimaliaHymenopteraTenthredinidae

Vikberg & Zinovjev, 2000

##### Diagnosis.

**Figure 1.**
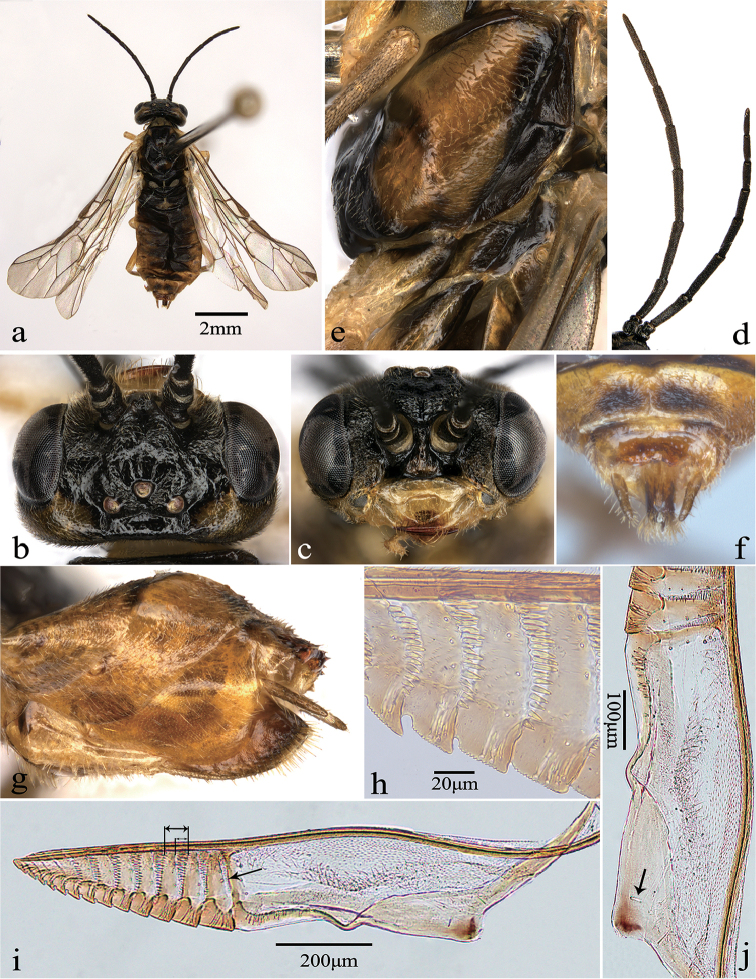
*Fagineuraflactoserrula* sp. n., female, holotype. **a** Dorsal view **b** head, dorsal view **c** head, anterior view **d** antenna, lateral view **e** mesopleuron and metapleuron **f** ovipositor sheath, dorsal view **g** ovipositor sheath, lateral view **h** middle serrulae **i** lancet; the short double arrow denotes the longest setae band, the long double arrow denotes the length of the annulus, the simple arrow denotes the annular suture 1 **j** tangium; the arrowhead denotes a single campaniform sensillum.

**Figure 2.**
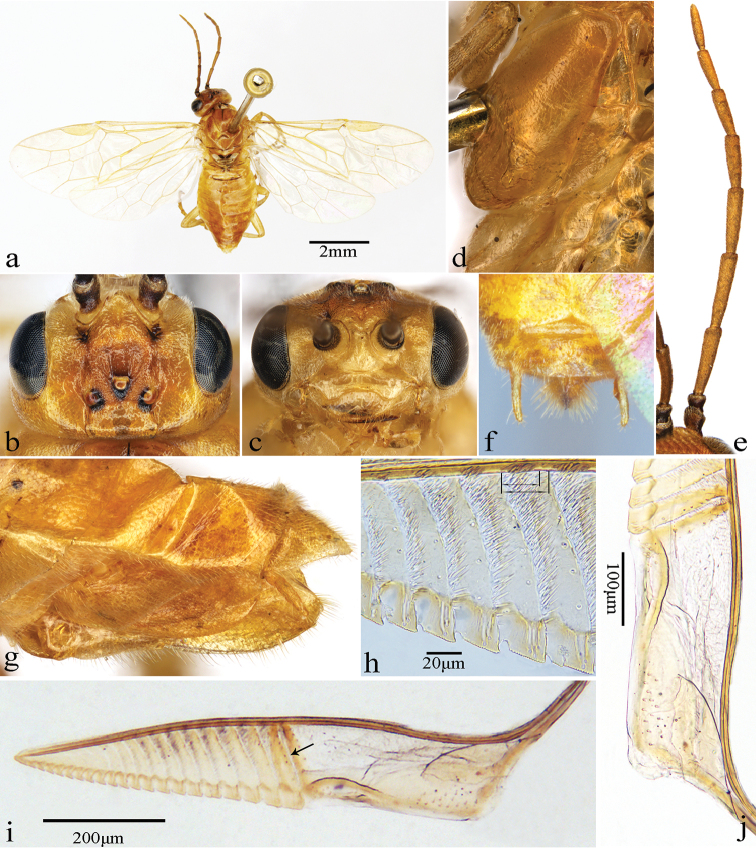
*Fagineuraxanthosoma* sp. n., female, paratype. **a** Dorsal view **b** head, dorsal view **c** head, anterior view **d** mesopleuron and metapleuron **e** antenna, lateral view **f** ovipositor sheath, dorsal view **g** ovipositor sheath, lateral view **h** middle serrulae; the short double arrow denotes the longest setae band, the long double arrow denotes the length of the annulus **i** lancet; the arrow denotes the annular suture 1 **j** tangium.

Medium-sized; clypeus and labrum yellowish-white to yellow; clypeus with broad and moderately deep (0.4–0.5) emargination apically; mandibles symmetrical; malar space shorter than diameter of median ocellus, and in most species not exceeding 0.5 × of diameter of median ocellus; postocellar area short, more than 2.0 × as wide as long; antenna usually shorter than thorax and abdomen together; posterior part of mesopleural katepimeron covered with hairs; distance between cenchri almost as long as breadth of a cenchrus; forewing without radial cross-vein; the costa of forewing less dilated than in *Pristiphora*; hindwing with anal cell petiolate; claws bifid, inner tooth large; sawsheath short; annular suture 1 with setae band; the longest setae bands of lancet is at least 0.5 × length of annulus (Figs 1i, 2h); cypsella of basal serrulae almost absent, apically short and with somewhat deep emargination; tangium of lancet with campaniform sensilla in most species; radix at least 0.5 × as long as lamnium, in most species radix not shorter than lamnium.

##### Remarks.

The genus resembles *Pristiphora*, *Mesoneura*, *Euura* and *Nematus*, but *Fagineura* can be distinguished from *Pristiphora* by having an emarginate clypeus; less dilated costa of the forewing; claws with a large inner tooth; in males, the posterior end of tergum 8 with distinct apical projection; distinguished from *Mesoneura* by the lack of radial cross-veins; apex of vein C in forewing slightly enlarged; abdomen longer than the head and thorax together; ovipositor sheath longer than fore tibia; distinguished from *Euura* and *Nematus* by an annular suture 1 with setae band; malar space narrower than the diameter of the median ocellus; katepimeron of the mesopleuron with hairs; having campaniform sensilla on the tangium in most cases.

### Key to species of *Fagineura* in the world

**Table:** 

1	Terga 1–2 black; lancet 14–15 serrulae	**2**
–	Terga 1–2 yellow; lancet 19–21 serrulae	**3**
2	Metapleuron pale yellowish; orbit yellowish to brownish in female; clypeus emarginated for about 0.5 of its length; postocellar area 2.5 × as wide as long; ovipositor sheath with shallow emargination apically; cerci reaching further back than sheath; annular suture 1 of lancet straight, and with 3 marginal sensilla below. Japan (Hokkaido, Honshu, Kyushu, Shikoku)	***F.crenativora* Vikberg & Zinovjev, 2000**
–	Metapleuron mostly black (Fig. 1e); orbit black in female (Fig. 1b–c); clypeus emarginated for about 0.3 of its length (Fig. 1c); postocellar area 3.0 × as wide as long (Fig. 1b); ovipositor sheath without emargination apically, cerci almost as long as sheath (Fig. 1f); annular suture 1 of lancet narrower on dorsal than on ventral side, with 7 marginal sensilla towards ventral side (Fig. 1i–j). China (Hubei)	***F.flactoserrula* sp. n.**
3	Mesepisternum entirely black; all coxae and apical 0.3 of hind tibia black; terga 3–10 mostly black; ovipositor sheath black; malar space nearly absent; petiole of anal cell of hindwing shorter than cu-a; tarsal claw with inner tooth longer than outer tooth; lancet with 19 serrulae. Japan (Honshu)	***F.quercivora* Togashi, 2006**
–	Mesepisternum entirely pale yellowish-brown (Fig. 2d); all coxae and hind tibia pale yellowish (Fig. 2a); terga 3–10 entirely pale yellowish-brown (Fig. 2a); ovipositor sheath yellow (Fig. 2g); malar space 0.8 times as long as diameter of median ocellus (Fig. 2c); petiole of anal cell of hindwing longer than cu-a (Fig. 2a); tarsal claw with inner tooth shorter than outer tooth; lancet with 21 serrulae (Fig. 2i). China (Hubei, Hunan)	***F.xanthosoma* sp. n.**

#### Fagineura flactoserrula sp. n.

Taxon classificationAnimaliaHymenopteraTenthredinidae

http://zoobank.org/00DF12C3-1549-4C0C-8B7E-80801376E2E0

Fig. 1

##### Type material.

**Holotype**, ♀, **China**, Hubei Province, Yichang City, Shennongjia Mountain, Yinyuhe, 31°34'00"N, 110°20'22"E, 2100m, 17 May 2012, leg. Zejian Li, CSCS. **Paratype**, 1♀, **China**, Hubei Province, Yichang City, Shennongjia Mountain, Yinyuhe, 31°34'00"N, 110°20'22"E, 2100 m, 16 May 2012, leg. Zejian Li.

##### Diagnosis.

Body mostly black; labrum and clypeus pale yellow (Fig. 1b–c); most parts of mesepisternum yellowish-brown (Fig. 1e); most of stigma pale yellowish-brown but margins black brown, veins in most part black brown; labrum and clypeus smooth and shiny, with few faint setigerous punctures, without microsculpture; frons slightly shiny, with hair warts and few wrinkles, punctures minute and sparse (Fig. 1b–c); vertex and postocellar area shiny, punctures faint and sparse, without microsculpture; malar space 0.5 × as long as diameter of median ocellus; postocellar area slightly convex, without mesosulcus, approx. 3.0 × as wide as long; relative length of antennomere 3 : antennomere 4 : antennomere 5 = 1.0 : 1.5 : 1.2 (Fig. 1d); forewings with cross-vein cu-a joining cell 1M at basal 0.5, cell 2Rs 1.2 × as long as wide, petiole of anal cell of hindwing 1.6 × as long as cu-a; lancet with 14 serrulae (Fig. 1i); annular suture 1 oblique and slightly curved, sutures 1–10 with setae bands, longest setae band about 0.7× length of annulus; tangium 3.4 × as long as annulus 1, radix 1.1 × as long as lamnium (Fig. 1j).

##### Description.

Holotype, female. Body length approximately 6.5 mm (Fig. 1a).

*Color.* Body mostly black. Labrum, clypeus, most parts of pronotum, most parts of propleuron, tegula, most parts of all coxae, all trochanters and femora pale yellow; most parts of vertex and temple, triangular spot of median mesoscutal lobe and mesoscutellum, most parts of mesepisternum, speckles on terga, sterna of abdomen, all tibiae and tarsi yellowish-brown; valvifer 2 pale yellow, valvula 3 yellowish-brown to black; cenchrus yellowish-white. Wings hyaline, most parts of stigma pale yellowish-brown with margins black brown, veins in most part black brown.

*Head.* Inner margins of eyes slightly convergent downward in frontal view, distance between eyes 1.9 × as long as height of eyes. Base of labrum elevated, apex slightly rounded; base of clypeus elevated, anterior margin of clypeus incised to 0.3 × length of clypeus, lateral corners rounded; labrum and clypeus shiny, with few faint setigerous punctures, without microsculpture. Malar space 0.5 × as long as diameter of median ocellus (Fig. 1c). Middle fovea long and groove-like, narrow and deep. Frons elevated, slightly shiny, with hair warts and few wrinkles, punctures minute and sparse; anterior wall slightly elevated and curved, notched medially, lateral walls low and blunt. Interocellar furrow broad and shallow, postocellar furrow slightly narrow and deep; circumocellar furrow indistinct; POL : OOL : OCL = 1.0 : 1.1 : 0.6 (Fig. 1b). Vertex and postocellar area shiny, punctures faint and sparse, without microsculpture; postocellar area slightly convex, without mesosulcus, approx. 3.0 × as wide as long, lateral furrows broad and slightly deep, parallel; in dorsal view, inner margins of eyes slightly divergent (Fig. 1b). Antenna filamentous, antennomere 3 slightly compressed, slightly shorter than thorax and abdomen together; antennomere 2 1.3 × as wide as long, relative length of antennomere 3 : antennomere 4 : antennomere 5 = 1.0 : 1.5 : 1.2 (Fig. 1d).

*Thorax.* Mesonotum shiny, with fine and slightly dense punctures, without microsculpture; median mesoscutal groove shallow and thin; mesoscutellum shiny, with faint and sparse punctures, and flat, posterior half of middle ridge distinct, about 0.8 × as long as wide; mesoscutellar appendage slightly shiny, with weak and sparse punctures, microsculpture faint, about 0.3 × length of scutellum, middle ridge low and blunt. Distance between cenchri as long as breadth of a cenchrus. Mesepisternum smooth and shiny, setigerous punctures and microsculpture indistinct; anepimeron of mesepimeron slightly shiny, with few wrinkles, punctures faint; katepimeron shiny, most parts with microsculpture and posterior part distinct, punctures weak and very sparse, posterior part covered with few setae; metapleuron shiny and smooth, with few weak punctures, microsculpture indistinct (Fig. 1e). Vein Sc interstitial with origin of vein M from R, and vein M slightly shorter than vein R+M; forewings with cross-vein cu-a joining cell 1M at basal 0.5, cell 2Rs 1.2 × as long as wide, petiole of anal cell of hindwing 1.6 × as long as cu-a.

*Abdomen.* All abdominal terga shiny, with faint sparse setigerous punctures, microsculpture fine and very dense. Ovipositor sheath smooth and shiny, punctures laterally on valvula 3 weak and sparse, microsculpture indistinct; sheath 2.0 × as long as metatarsomere 1 and 1.3 × as long as front tibia, valvula 3 as long as valvifer 2; in lateral view, sheath tapering toward apex (Fig. 1g); in dorsal view, apex of cercus protruding beyond valvula 3, angle between most lateral setae of valvula 3 about 60° (Fig. 1f). Lancet with 14 serrulae (Fig. 1i); each middle serrula with 10–13 distal teeth (Fig. 1h); annular suture 1 oblique and slightly curved, sutures 1–10 with setae bands, longest setae band about 0.7 × length of annulus; cypsella of serrulae 1–5 nearly absent, cypsella of serrulae 6–12 short and deep; tangium 3.4 × as long as annulus 1, with one campaniform sensillum (Fig. 1j), radix 1.1 × as long as lamnium.

*Legs.* Protarsomere 1 shorter than combined length of tarsomeres 2–4; inner apical spur of hind tibia 0.4 × as long as metatarsomere 1, metatarsomere 1 0.6 × as long as combined length of metatarsomeres 2–5; tarsal claw with inner tooth long, but slightly shorter than outer tooth.

**Male**. Unknown.

##### Distribution.

China (Hubei).

##### Variation.

Triangular spot on median mesoscutal lobe yellowish-brown to black-brown; mesoscutellum entirely black, or sometimes with yellowish-brown speckles; tangium with one campaniform sensillum, or none.

##### Remarks.

The new species is similar to *F.crenativora* Vikberg & Zinovjev, 2000, but can be distinguished from the latter by the following characters: metapleuron mostly black; orbit black in the female; postocellar area 3.0 × as wide as long; sheath without emargination apically; cerci almost as long as the sheath; lancet with 14 serrulae, annular suture 1 of lancet oblique and slightly curved, and with 7 marginal sensilla below.

##### Etymology.

The specific name is derived from the flattened serrulae of the lancet.

#### Fagineura xanthosoma sp. n.

Taxon classificationAnimaliaHymenopteraTenthredinidae

http://zoobank.org/761893DD-B1DA-4A46-A692-85FF34DC7EAD

Fig. 2

##### Type material.

**Holotype**, ♀, **China**, Hubei Province, Yichang City, Shennongjia Mountain, Yinyuhe, 31°34'00"N, 110°20'22"E, 2100m, 17 May 2012, leg. Zejian Li, CSCS. **Paratypes** (15♀♀): 1♀, **China**, Hubei Province, Yichang City, Shennongjia Mountain, Yinyuhe, 31°34'00"N, 110°20'22"E, 2100 m, 17 May 2012, leg. Zejian Li; 1♀, **China**, Hunan Province, Wugang City, Yun Mountain, Television tower, 26°38'38"N, 110°37'18"E, 1380 m, 11 April 2012, leg. Zejian Li and Zaiyang Pan; 1♀, **China**, Hunan Province, Wugang City, Yun Mountain ,1100 m, 25 April 2005, leg. Yingke He; 8♀♀, **China**, Hunan Province, Yongzhou City, Yangming Mountain, 900 to 1000 m, 24 April 2004, leg. Shaobing Zhang; 3♀♀, **China**, Hunan Province, Yongzhou City, Yangming Mountain, 1000 to 1300 m, 24 April 2004, leg. Meicai Wei; 1♀, **China**, Hunan Province, Yongzhou City, Yangming Mountain, 1000 to 1300 m, 24 April 2004, leg. Wei Xiao.

##### Diagnosis.

Body pale yellow to pale yellowish-brown; stigma and most parts of veins pale yellow (Fig. 2a); frons slightly shiny, with some hair warts and wrinkles, punctures weak and very sparse; malar space 0.8 × as long as diameter of median ocellus; interocellar furrow broad and very shallow, postocellar furrow broad and slightly shallow; postocellar area convex, without mesosulcus, 2.8 × as wide as long (Fig. 2b–c); relative length of antennomere 3 : antennomere 4 : antennomere 5 = 1.0 : 1.2 : 1.0 (Fig. 2e); vein M about as long as vein R+M; forewings with cross-vein cu-a joining cell 1M at basal 0.6, cell 2Rs 1.4 × as long as wide, petiole of hind anal cell 1.3 × as long as cu-a (Fig. 2a); lancet with 21 serrulae (Fig. 2i); each middle serrula always with 14–17 distal teeth (Fig. 2h); annular suture 1 straight but oblique, sutures 1–13 with setae bands, longest setae band approx. 0.9 length of annulus; tangium 5.5 × as long as annulus 1, radix 0.6 × as long as lamnium (Fig. 2i, 2j).

##### Description.

Holotype, female. Body length approximately 7.0 mm (Fig. 2a).

*Color.* Body pale yellow to pale yellowish-brown. Lateral fovea, around ocelli, dorsal side of scape and pedicel, anterior edge and medial spot of tergum 1 black; cenchrus yellowish-white. Wings hyaline, stigma and most parts of veins pale yellow.

*Head.* Inner margins of eyes slightly convergent downward in frontal view, distance between eyes 2.4 × as long as height of eye (Fig. 2c). Base of labrum elevated, and apex rounded; base of clypeus elevated, anterior margin of clypeus incised to 0.3 × length of clypeus, lateral corners rounded; labrum and clypeus smooth and shiny, with few faint setigerous punctures, without microsculpture. Malar space 0.8 × as long as diameter of median ocellus (Fig. 2c). Middle fovea long, groove-like, deep. Frons slightly elevated, slightly shiny, with some hair warts and wrinkles, punctures weak and very sparse; anterior wall elevated and curved, notched medially, lateral walls distinct, but low and blunt. Interocellar furrow broad and very shallow, postocellar furrow broad and shallow; circumocellar furrow indistinct; POL : OOL : OCL = 0.9 : 1.0 : 0.7 (Fig. 2b). Vertex and postocellar area shiny, punctures faint and sparse, microsculpture indistinct; postocellar area convex, without mesosulcus, 2.8 × as wide as long, lateral furrows short, slightly broad and shallow; in dorsal view, inner margins of eyes subparallel (Fig. 2b). Antenna filiform, shorter than thorax and abdomen together, antennomere 3 slightly compressed; antennomere 2 1.3 × as wide as long, relative length of antennomere 3 : antennomere 4 : antennomere 5 = 1.0 : 1.2 : 1.0 (Fig. 2e).

*Thorax.* Mesonotum slightly shiny, with minute and dense punctures, microsculpture indistinct; median mesoscutal groove shallow and narrow; mesoscutellum shiny, with weak and slightly sparse punctures, and flat, middle ridge indistinct, 0.8 × as long as wide; mesoscutellar appendage shiny, with faint and sparse punctures, without microsculpture, approx. 0.4 × as long as scutellum, middle ridge faint. Distance between cenchri as long as breadth of cenchrus. Mesepisternum shiny, setigerous punctures weak and slightly dense, microsculpture indistinct; mesepimeron shiny, with few faint punctures, with some microsculpture on margins, posterior part of katepimeron extensively covered with setae; metapleuron shiny and smooth, punctures and microsculpture indistinct (Fig. 2d). Vein Sc little basad of origin of vein M from R, vein M about as long as vein R+M; forewings with cross-vein cu-a joining cell 1M at basal 0.6, cell 2Rs 1.4 × as long as wide, petiole of anal cell of hindwing 1.3 × as long as cu-a (Fig. 2a).

*Abdomen.* All abdominal terga slightly shiny, with faint and sparse setigerous punctures, microsculpture fine and very dense. Ovipositor sheath shiny, punctures laterally on valvula 3 weak and sparse, microsculpture indistinct; ovipositor sheath 1.9 × as long as metatarsomere 1 and 1.2 × as long as front tibia, valvula 3 1.3 × as long as valvifer 2; in lateral view, sheath tapering toward apex (Fig. 2g); in dorsal view, apex of cercus protruding beyond valvula 3, angle between most lateral setae of valvula 3 about 85° (Fig. 2f). Lancet with 21 serrulae (Fig. 2i); each middle serrula always with 14–17 distal teeth (Fig. 2h); annular suture 1 straight but oblique, sutures 1–13 with setae bands, longest setae band approx. 0.9 × length of annulus; cypsella of serrulae 1–2 nearly absent, cypsella of serrulae 3–19 very short and deep; tangium 5.5 × as long as annulus 1, with many campaniform sensilla (Fig. 2j), radix 0.6 × as long as lamnium (Fig. 2i).

*Legs.* Protarsomere 1 slightly shorter than combined length of tarsomeres 2–4; inner apical spur of hind tibia 0.4 × as long as metatarsomere 1, metatarsomere 1 0.7 × as long as combined length of metatarsomeres 2–5; tarsal claw with inner tooth slightly shorter than outer tooth.

**Male**. Unknown.

##### Distribution.

China (Hubei, Hunan).

##### Variation.

Body length 6.0–8.0mm; scape and pedicel partly to entirely black; around ocelli more or less black; vein Sc a little basad or interstitial with origin of vein M from R, and vein M as long as or slightly shorter than vein R+M; petiole of anal cell of hindwing 1.2–1.6 × as long as cu-a; in dorsal view, apex of cercus protruding far as or beyond valvula 3.

##### Remarks.

The new species is similar to *F.quercivora* Togashi, 2006, but can be distinguished from the latter by the following characters: mesepisternum entirely pale yellowish-brown; all coxae and hind tibia pale yellowish; terga 3–10 entirely pale yellowish-brown; ovipositor sheath yellow; malar space 0.8 × as long as diameter of median ocellus; petiole of anal cell of hindwing longer than cu-a; tarsal claw with inner tooth shorter than outer tooth; lancet with 21 serrulae.

##### Etymology.

The specific name is derived from the body color of adults.

### Phylogenetic analyses

**Figure 3.**
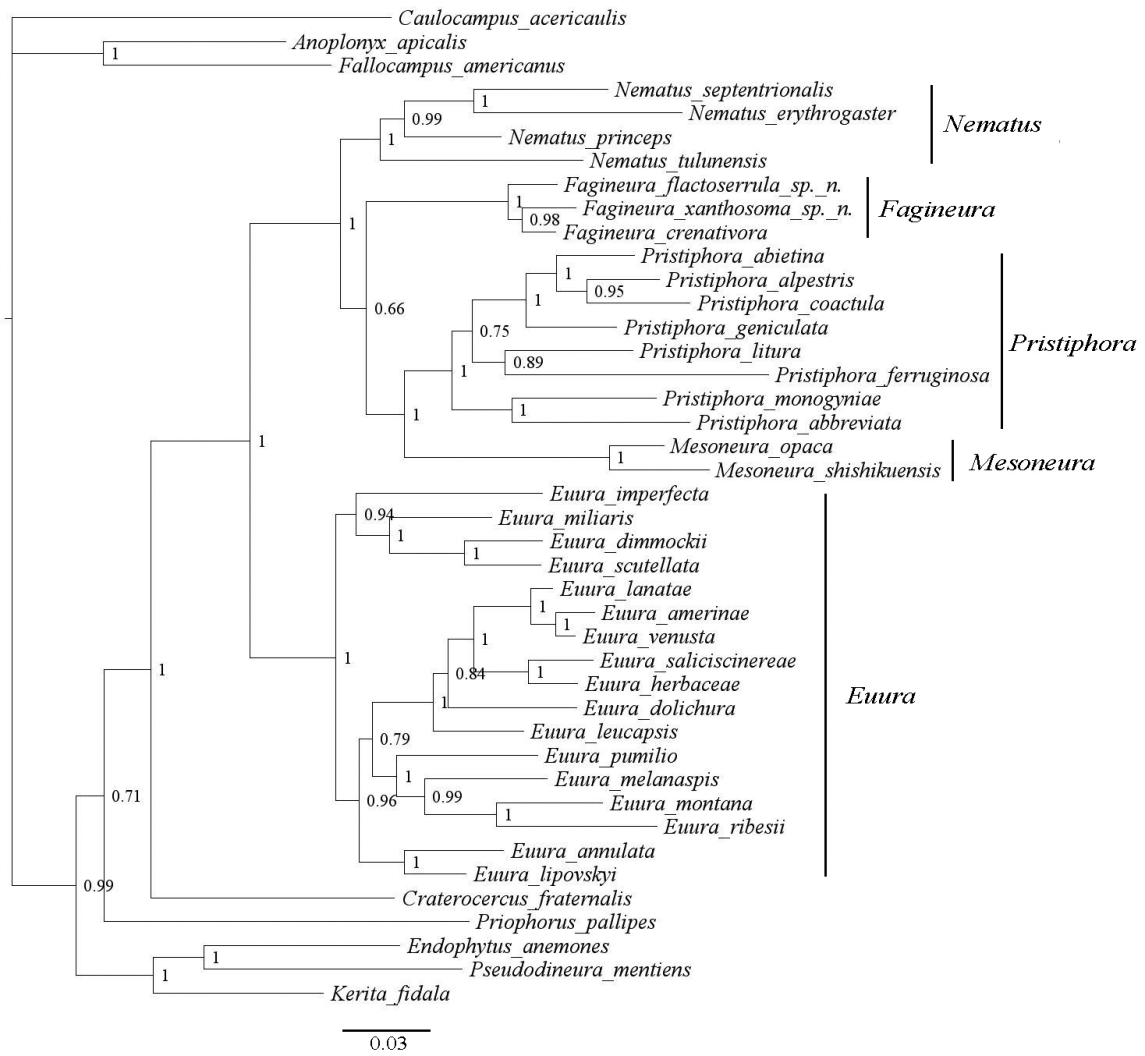
Phylogenetic tree of three *Fagineura* species and other representative species of the Nematinae based on Bayesian phylogenetic analysis of COI and NaK sequences. Numbers at right of nodes show Bayesian posterior probabilities (PP). The scale bar shows the number of estimated substitutions per nucleotide position.

A Kimura 2-parameter model of COI and NaK distances within *Fagineura* species is shown in Table 2, and the mean distances within *Nematus*, *Fagineura*, *Pristiphora*, *Euura*, *Mesoneura* respectively and distances between these genera are shown in Table 3. The best-fitting model for the two-gene alignment was GTR+I+G (Nei and Kumar 2000). In MrBayes, default priors were used, and two parallel runs having four incrementally heated chains for 1.5 million generations were made, while sampling trees from the current cold chain every 1000 generations. 375 trees sampled were discarded prior to reaching chain stationarity as a burn-in from both runs, and the remaining 1126 trees were used to calculate a 50% majority consensus rule tree, showing all groupings with posterior probability more than 0.5 (Fig. 3).

**Table 2.** Kimura 2-parameter model distances among *Fagineura* species based on COI (below) and NaK (above) sequences.

Species	Distance between species		
	* F. flactoserrula *	* F. xanthosoma *	* F. crenativora *
* Fagineura flactoserrula *		0.005	0.005
* Fagineura xanthosoma *	0.067		0.004
* Fagineura crenativora *	0.052	0.050	

The two new species and *Fagineuracrenativora* are separated by an adequate distance (Tables 2, 3), and all three species form a monophyletic group (Fig. 3). The K2P distance based on COI and NaK between *F.flactoserrula* and *F.xanthosoma* are 6.7% and 0.5%, between *F.flactoserrula* and *F.crenativora* 5.2% and 0.5%, and between *F.xanthosoma* and *F.crenativora* 5.0% and 0.4%, respectively. The distance, based on COI and NaK, is 11.6% between *Fagineura* and *Mesoneura* and 8.9% between *Fagineura* and *Nematus*. These results are consistent with the morphological taxonomy described above. Unfortunately, sequences of *F.quercivora* are not available in GenBank, and we did not have any specimens of this species available. However, the two new species can be easily separated from *F.quercivora* by morphological characters.

**Table 3.** Mean Kimura 2-parameter model distances for COI (below) and NaK (above) within and among large genera of the Nematinae.

Genus	Distance within genus		Distance between genera				
	COI	NaK	* Nematus *	* Fagineura *	* Pristiphora *	* Euura *	* Mesoneura *
* Nematus *	0.140	0.052		0.089	0.090	0.110	0.108
* Fagineura *	0.056	0.005	0.123		0,091	0.119	0.109
* Pristiphora *	0.139	0.042	0.148	0.128		0.129	0.106
* Euura *	0.111	0.052	0.135	0.121	0.148		0.154
* Mesoneura *	0.062	0.026	0.134	0.116	0.131	0.129	

## Discussion

In this paper, two new species of *Fagineura* are described and illustrated. Compared to the generic characters of *Fagineura* proposed by Shinohara et al. (2000) and Prous et al. (2014), there are two differences in *F.flactoserrula*, including that the tangium lacks or has only one campaniform sensillum, and that the mesothoracic katepimeron is covered with only few setae. However, the generic characterisation in the earlier publications was based only on the two species known at that time, so that the previous definition of the genus apparently does not encompass the full range of interspecific variability. The phylogenetic analyses support placement of the two new species in *Fagineura*, and that they are different from *F.crenativora*. The new species are also different from *F.quercivora* based on morphological characters.

## Supplementary Material

XML Treatment for Fagineura

XML Treatment for Fagineura flactoserrula

XML Treatment for Fagineura xanthosoma

## References

[B1] Benson RB (1954) Some sawflies of the European Alps and the Mediterranean region (Hymenoptera: Symphyta). Bulletin of the British Museum (Natural History). Entomology series 3(7): 267–295.

[B2] Darriba D Taboada GL Doallo R Posada D (2012) jModelTest 2: more models, new heuristics and parallel computing. Nature Methods 9: 772. 10.1038/nmeth.2109 PMC4594756 22847109

[B3] Guindon S Gascuel O (2003) A simple, fast, and accurate algorithm to estimate large phylogenies by maximum likelihood. Systematic Biology 52: 696–704. 10.1080/10635150390235520 14530136

[B4] Kumar S Stecher G Tamura K (2016) MEGA7: Molecular evolutionary generics analysis Version 7.0 for bigger datasets. Molecular Biology and Evolution 33(7): 1870–1874. 10.1093/molbev/msw054 27004904 PMC8210823

[B5] Leppänen SA Altenhofer E Liston AD Nyman T (2012) Phylogenetics and evolution of host-plant use in leaf-mining sawflies (Hymenoptera: Tenthredinidae: Heterarthrinae). Molecular Phylogenetics and Evolution 64: 331–341. 10.1016/j.ympev.2012.04.005 22531610

[B6] Nei M Kumar S (2000) Molecular Evolution and Phylogenetics. Oxford University Press, New York.

[B7] Normark BB Jordal BH Farrell BD (1999) Origin of a haplodiploid beetle lineage. Proceedings of the Royal Society B 266: 2253–2259. 10.1098/rspb.1999.0916

[B8] Nyman T Zinovjev AG Vikberg V Farrell B (2006) Molecular phylogeny of the sawfly subfamily Nematinae (Hymenoptera: Tenthredinidae). Systematic Entomology 31: 569–583. 10.1111/j.1365-3113.2006.00336.x

[B9] Prous M Blank SM Goulet H Heibo E Liston A Malm T Nyman T Schmidt S Smith DR Vardal H Viitasaari M Vikberg V Taeger A (2014) The genera of Nematinae (Hymenoptera,Tenthredinidae). Journal of Hymenoptera Research 40: 1–69. 10.3897/JHR.40.7442

[B10] Prous M Kramp K Vikberg V Liston AD (2017) North-Western Palaearctic species of *Pristiphora* (Hymenoptera, Tenthredinidae). Journal of Hymenoptera Research 59: 1–190. 10.3897/Jhr.59.12656

[B11] Ronquist F Teslenko M van der Mark P Ayres DL Darling A Höhna S Larget B Liu L Suchard Ma Huelsenbeck JP (2012) MrBayes 3.2: efficient Bayesian phylogenetic inference and model choice across a large model space. Systematic Biology 61: 539–542. 10.1093/sysbio/sys029 22357727 PMC3329765

[B12] Ross HH (1945) Sawfly genitalia: terminology and study techniques. Entomological News 61(10): 261–268.

[B13] Shinohara A Vikberg V Zinovjev AG Yamagami A (2000) *Fagineuracrenativora*, a New Genus and Species of Sawflies (Hymenoptera, Tenthredinidae, Nematinae) Injurious to Beech Trees in Japan. Bulletin of the National Science Museum, Series A, Zoology 26(3): 113–124.

[B14] Taeger A Blank SM Liston AD (2010) World Catalog of Symphyta (Hymenoptera). Zootaxa 2580: 1–1064. 10.11646/zootaxa.2580.1.1

[B15] Takeuchi K (1952) A Generic Classification of the Japanese Tenthredinidae (Hymenoptera: Symphyta). Published by the authors’ friends, Kyoto, 90 pp.

[B16] Togashi I (2006) A new sawfly, *Fagineuraquercivora* (Hymenoptera: Tenthredinidae) feeding on *Quercusserrata* and *Q.mongolicacrispula* in Honshu, Japan. Proceedings of the Entomological Society of Washington 108(1): 169–173.

[B17] Viitasaari M (2002) The Suborder Symphyta of the Hymenoptera. In: Viitasaari M (Ed.) Sawflies (Hymenoptera, Symphyta) I.A review of the suborder, the Western Palaearctic taxa of Xyeloidea and Pamphilioidea. Tremex, Helsinki, 11–174.

